# Recent Advances in Degradation of Polymer Plastics by Insects Inhabiting Microorganisms

**DOI:** 10.3390/polym15051307

**Published:** 2023-03-05

**Authors:** Rongrong An, Chengguo Liu, Jun Wang, Puyou Jia

**Affiliations:** 1 School of Geographic and Biologic Information, Nanjing University of Posts and Telecommunications, Nanjing 210023, China; 2 Institute of Chemical Industry of Forest Products, Chinese Academy of Forestry, 16 Suojin North Road, Nanjing 210042, China

**Keywords:** plastic, biodegradation, polypropylene, polyethylene, polyvinyl chloride, insect

## Abstract

Plastic pollution endangers all natural ecosystems and living creatures on earth. Excessive reliance on plastic products and excessive production of plastic packaging are extremely dangerous for humans because plastic waste has polluted almost the entire world, whether it is in the sea or on the land. This review introduces the examination of pollution brought by non-degradable plastics, the classification and application of degradable materials, and the current situation and strategy to address plastic pollution and plastic degradation by insects, which mainly include *Galleria mellonella*, *Zophobas atratus*, *Tenebrio molitor*, and other insects. The efficiency of plastic degradation by insects, biodegradation mechanism of plastic waste, and the structure and composition of degradable products are reviewed. The development direction of degradable plastics in the future and plastic degradation by insects are prospected. This review provides effective ways to solve plastic pollution.

## 1. Introduction

### 1.1. Application and Pollution of Non-Degradable Plastics

Plastic products have been widely used around the world because of low cost and easy production. Polystyrene (PS), polyvinyl chloride (PVC), polyethylene (PE), acrylonitrile butadiene styrene (ABS), and polyurethane (PU), which are usually designed as short-term and disposable products, are commonly used plastics [1,2,3]. Plastic products have brought great convenience to people’s lives. However, a large number of plastic products have become the focus of global attention because of the environmental problems caused by their improper disposal after being used and discarded [4,5]. The global plastic output and consumption exceed 300 million tons annually, which has grown exponentially in the past 50 years [6,7]. In 27 EU countries, including Norway and Switzerland, 38% of plastic is discarded in landfills, whereas the rest is used for recycling (26%) and energy recovery through combustion (36%) [8]. The world’s disposable plastic products reach 120 million tons every year, only 10% of which are recycled, 12% are burned, and more than 70% are discarded into the soil, air, and sea [9,10]. More than one trillion plastic bags are consumed every year [11]. In recent years, microplastics, as a new kind of pollutant, have attracted more attention because of their widespread distribution in the oceans and coastal waters around the world, which have polluted the marine ecological environment and marine organisms [12]. Considering that many waste plastics have not been treated scientifically and correctly, they form microplastics through a series of physical and chemical processes and enter the ocean. Some animals, such as birds, fish, and sea turtles, have been affected by plastic pollution [13]. Many plastic particles were discovered in the intestines of dead birds, fish, and turtles, indicating that plastic has caused serious damage to living creatures and bodies [14]. Animals cannot distinguish food and plastic in the environment, resulting in ingestion of plastic particles. Plastics cannot be digested and often accumulate in the body. When plastics are decomposed in the marine environment, the micro plastics, microfibers, toxic chemicals, metals, and organic micro pollutants will be transferred to the waters and sediments and finally enter the marine food chain. These substances affect the reproductive success rate and viability of marine organisms and damage the ability of “ecological engineer” corals and worms in the aquatic ecosystem to build coral reefs and change sediments through biological disturbance.

### 1.2. Classification and Application of Degradable Plastics

The scientific disposal of discarded plastic products has become a challenge. Therefore, researchers exert their energy into the study of degradation plastics. Degradable plastics meet the use requirements during the storage period and can be decomposed into harmless substances under natural environmental conditions after use. It is considered an effective way to deal with plastic pollution and has attracted attention in recent years [15]. Many kinds of degradable plastic products are available on the market, and the product performance and capacity scale are also quite different [16]. Degradable plastics include biological and petrochemical plastics based on the source of raw materials. Bio-based degradable plastics include polylactic acid (PLA), polyhydroxy fatty acid ester polymers (PHAs), starch, and cellulose; and petrochemical-based degradable plastics include carbon dioxide copolymer (PPC), polycaprolactone (PCL), and polyglycolic acid (PGA) [17]. Among them, degradable plastics based on starch are gradually eliminated because they cannot be degraded completely. PBAT, PBS, and PLA have formed industrial-scale production in the market and occupied a large market share. From the specific use of various degradable plastics, PBAT/PBS products are mainly used in packaging, tableware, cosmetic bottles, disposable medical supplies, agricultural films, pesticides, and fertilizer slow-release materials [18]. PCL is used in the production of toys, bone nails, long-acting drugs, and in other medical fields. PLA is used in general plastics, such as films, lunch boxes, and textiles. PPC is used in film bag, surgical suture, bone nail and other medical fields, membrane, and packaging materials. The global degradable plastic industry is at the initial stage of industrialization, and China’s production capacity has increased significantly. In the past five years, the average growth rate of the consumption of biodegradable plastics in China was at 20% [16]. In 2019, the consumption of biodegradable plastics in China was approximately 260,000 tons. The estimated demand for biodegradable plastics in China will exceed 650,000 tons by 2024 [19].

### 1.3. Current Situation and Strategy to Address Plastic Pollution

Hundreds of years are required for plastics to be degraded naturally, including 600 years for PE and PP plastics, which will occupy land for a long time, affect crops and livestock, and cause serious white pollution [20]. A large amount of plastic waste flows into the sea after being generated. According to statistics, 3% (approximately 11 million tons in 2019) of plastic waste enters the sea every year. Approximately 14 million tons of micro plastics, which seriously affect the safety and health of marine organisms, can be found in the whole sea floor. The main disposal methods of waste plastics include landfill, incineration, chemical treatment, and recycling. Landfill method destroys soil, affects groundwater, and cannot effectively degrade waste plastics. Incineration produces a large number of toxic gases, which volatilize into the air and affect human health. The cost of chemical treatment and recycling is high. Hence, it is not suitable for wide use. Plastic waste residues do not decompose in soil for a long time, thereby damaging the physical and chemical properties of the soil, affecting the growth of plant roots and leading to crop production reduction. Waste plastics cause water pollution. According to the World Greenpeace Organization in 2016, more than 200 kg of plastics are dumped into the sea every second in the world [20,21]. The concentration of plastic fragments observed in the oceans around the world is as high as 58,000 per square kilometer. Approximately millions of marine animals die every year due to suffocation or indigestion caused by consumption of plastic by mistake. The United States is a major plastic producer in the world, and it had carried out research on the recycling of waste plastics in 1960s. The recycling rate of waste plastics exceeded 35% at the beginning of the 21st century, the average recycling rates of plastics in Europe and Germany exceeded 45% and were as high as 60% [22]. In Japan, 52% of waste plastics were recycled, of which 2% were used as chemical raw materials, 3% as remelted solid fuel, 20% as power generation fuel, and 43% as heat energy in incinerators.

Plastic pollution has endangered the natural ecosystems and living creatures on the earth, as well as human health. Recent reports on the plastic degradation by insects have drawn widespread attention (Table 1). These insects mainly include *Galleria mellonella*, *Zophobas atratus*, *Tenebrio molitor*, and other insects. In the review, the efficiency of plastic degradation by insects, biodegradation mechanism of plastic waste, and the structure and composition of degradable products are reviewed. The development direction of degradable plastics in the future and plastic degradation by insects are prospected. These degradation strategies and results provide effective ways to solve the issue of plastic pollution.

## 2. Plastic Degradation by Insects

### 2.1. Plastic Degradation by T. molitor

*T. molitor* is a completely metamorphosed insect [46,47]. The larvae of *T. molitor* like to flock, live, and feed at 13 °C. They can still grow at temperatures above 35 °C but cannot survive at temperatures above 50 °C. *T. molitor* can be used as an excellent feed for medicinal animals, such as scorpions, centipedes, and fish. *T. Molitor* can also be used as food raw materials, health products, and medicine for human beings [48]. 

Recently, the study on plastic degradation by *T. molitor* has been widely districuted [23,24,25,26,28]. Intestinal microbes of *T. molitor* larvae play a decisive role in the biodegradation of PS. The study reported that the larvae cannot degrade plastics after adding antibiotics to the food of *T. molitor* larvae to inhibit intestinal microorganisms. The researchers further successfully isolated the PS degrading bacterium exiguobacterium sp. (YT2), which can grow by using PS as the only carbon source. This strain of bacteria can grow on the surface of PS film on carbon-free agar solid medium to form a stable biofilm that can significantly erode the surface structure of the film. After the strain was cultured in liquid for 60 days, the PS chips in the liquid were decomposed, and the weight loss was up to 7.4%. The molecular weight of the residue decreased significantly, and a large number of water-soluble low-molecular intermediates were generated. Microorganisms can degrade PS. This study provides a new way to develop biodegradable polymer materials and waste plastic treatment technology [23,24]. To study the ability of *T. molitor* larvae to degrade PE and the depolymerization mode, Shan Shan Yang et al. fed two commercial low-density PS (LDPE) foam to *T. molitor* larvae at ambient temperature within 36 days. The residual PE in the feces of *T. molitor* larvae decreased by 43.3% and 31.7 ± 0.5%. The structure shows that low molecular weight PE (<5.0 kDa) is rapidly digested, whereas the long chain part (>10.0 kDa) is decomposed or cracked, indicating a widespread depolymerization. Mass balance analysis showed that 40% of ingested LDPE was digested into CO_2_ [25]. Craig S. Criddle et al. discovered eight unique intestinal microorganisms related to PS biodegradation, including *Citrobacter freundii*, *Serratia marcescens*, and *Klebsiella aerogenes*. The intestinal microbes of *T. molitor* were helpful in accelerating the plastic biodegradation. This work provides a potential strategy for future research and large-scale cultivation of plastic-degrading microorganisms [26]. 

**Figure 1:**
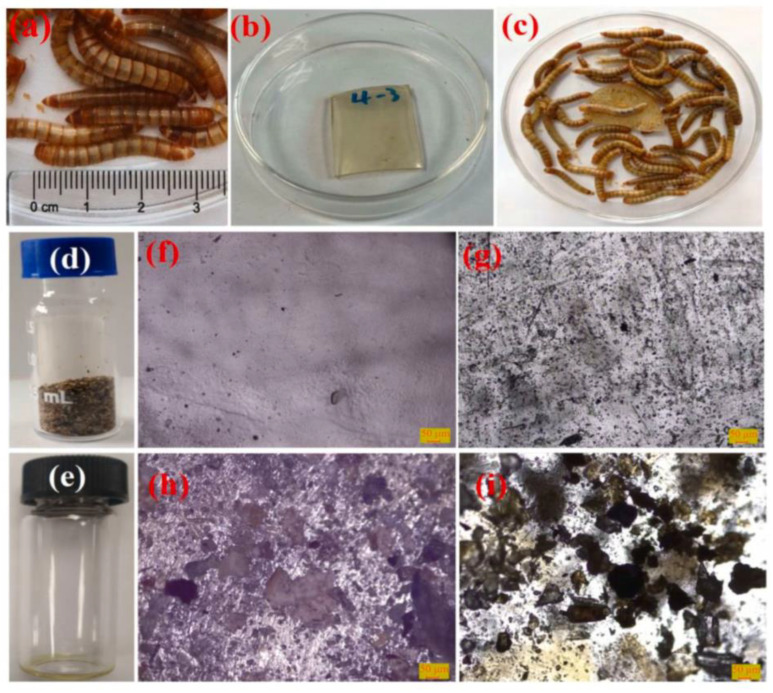
(**a**) Representative images of *Tenebrio molitor*; (**b**) bio-based cross-linked polymer film used in biodegradation experiment; (**c**) Tenebrio monitor fed with bio-based cross-linked polymer film for 30 days. (**d**) Feces of *Tenebrio molitor* collected in biodegradation experiment. (**e**) Extraction from feces of *Tenebrio molitor* using tetrahydrofuran (THF). (**f**) Microstructures of bio-based cross-linked polymer film in a reflection mode. (**g**) Microstructures of bio-based cross-linked polymer film in a transmissive mode. (**h**) Microstructure of bio-based cross-linked polymer films after feeding *Tenebrio molitor* for 30 days in a reflection mode. (**i**) Microstructures of bio-based cross-linked polymer 3 film after feeding *Tenebrio molitor* for 30 days in a transmissive mode, reprinted with permission from [30].

To determine whether plastics are sensitive to biodegradation in *T. molitor*, Craig S. Criddle et al. fed *T. molitor* with PE and PS, and the molecular weight (Mn) of polymer residues decreased by 40.1% and 12.8%, respectively [28]. Emmanouil Tsochatzis et al. investigated the intestinal microbes and the formation of degradation compounds of *T. molitor* larvae under different feeding strategies. The results showed that water can significantly improve the biodegradation of PS monomer and oligomer residues. Diet leads to differences in intestinal microbiota, and three potential bacterial strains were identified as the candidate strains involved in PS biodegradation [27]. Yalei Zhang et al. investigated the biodegradation of PVC by feeding *T. molitor* larvae with PVC micro plastic powder. After 16 days, the Mw, Mn, and Mz of PVC decreased by 33.4%, 32.8%, and 36.4%, respectively. The degradation products contain O-C and O-C functional groups. The survival rate of *T. molitor* larvae with PVC, as the sole food in 5 weeks, was as high as 80%, and the survival rate in three months was as low as 39%. PVC and wheat bran were fed together to *T. molitor*, and they completed the growth and pupation process within 91 days. *T. molitor* larvae can degrade PVC, but the mineralization of PVC is limited [29]. Yonghong Zhou et al. reported that *T. molitor* could degrade bio-based cross-linked polymers. Figure 1a–i shows representative images of *T. Molitor*, bio-based cross-linked polymer film used in biodegradation experiment, and biodegradation test results. A total of 8% (0.2 g) of polymer was biodegraded in the digestive system of *T. molitor*. The degradation products contain products with low molecular weight, which is the result of chain break caused by microbial attack. Biodegradation showed that the prepared biopolymer network has good degradation performance and better impact on the environment [30]. Piotr Bulak et al. studied the ability of *T. molitor* to biodegrade PE, PU, and PS foams. The results showed that the quality of PS, PU1, PU2, and PE decreased by 46.5%, 41.0%, 53.2%, and 69.7% after 58 days, which indicated that the larvae and adults of *T. molitor* could degrade plastic [31].

### 2.2. Plastic Degradation by Z. atratus 

*Z. atratus* are mainly distributed in Central and South America, West Indies and other places, which are also known as super bread worms and super wheat barkworms [49,50]. The maximum body length of adult larvae is approximately 71 cm, which is 3–4 times larger than that of the general yellow mealworm, and the yield is 5 times higher than that of yellow mealworm. *Z. atratus* larvae are rich in protein and fat, of which the protein content can reach more than 51%, and the fat content is approximately 29%. At the same time, they also contain many nutrients, including sugars, amino acids, vitamins, and minerals. *Z. atratus* can degrade plastic similarly to *T. molitor*.

**Figure 2:**
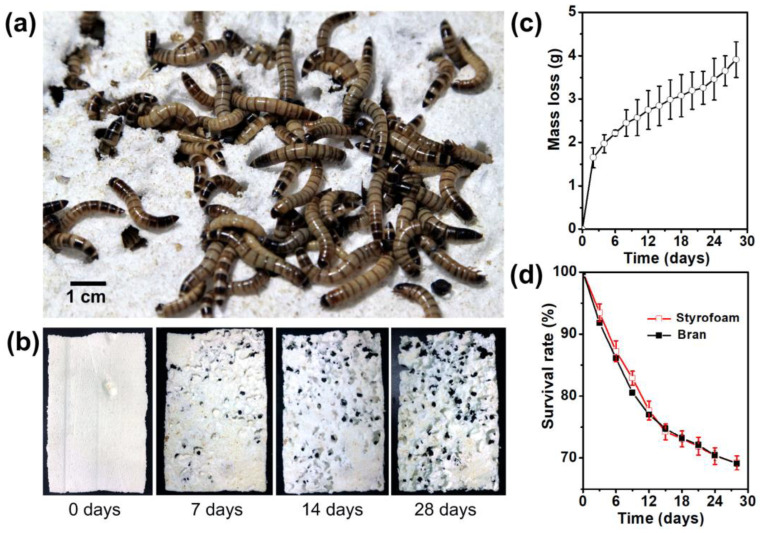
PS foam-eating activities of *Zophobas atratus*. (**a**) *Zophobas atratus* like to eat and penetrate PS foam. (**b**) Increasing hollows in the Styrofoam block; (**c**) consumption of PS foam by a group of *Zophobas atratus*; (**d**) survival rate of PS foam-eating and normal diet (bran)-eating *Zophobas atratus,* reprinted with permission from [32].

Yu Yang et al. determined that *Z. atratus* could ingest PS as the only food or could be fed with bran for 28 days. Figure 2a–d shows the PS foam-eating activities of *Z. atratus*, the increasing hollows in the Styrofoam block, consumption of PS foam by a group of *Z. atratus*, and survival rate of PS foam-eating and normal diet (bran)-eating *Z. atratus*. The results showed that the average consumption rate of PS foam plastic of each superworm is estimated to be 0.58 mg/d, which is four times than that of *T. molitor*. The depolymerization of long-chain PS molecules and the formation of low molecular weight products occurred in the gut of larvae. During the 16-day test period, up to 36.7% of the intake of foam plastic carbon was converted into CO_2_. Antibiotics of intestinal microbiota inhibited the degradation of PS by *Z. atratus*, indicating that intestinal microbiota contributed to PS degradation. This new discovery extends PS degrading insects beyond Tenebrio species and indicates that the intestinal microbiota of *Z. atratus* will be a new biological source for plastic-degrading enzymes [32].

**Figure 3:**
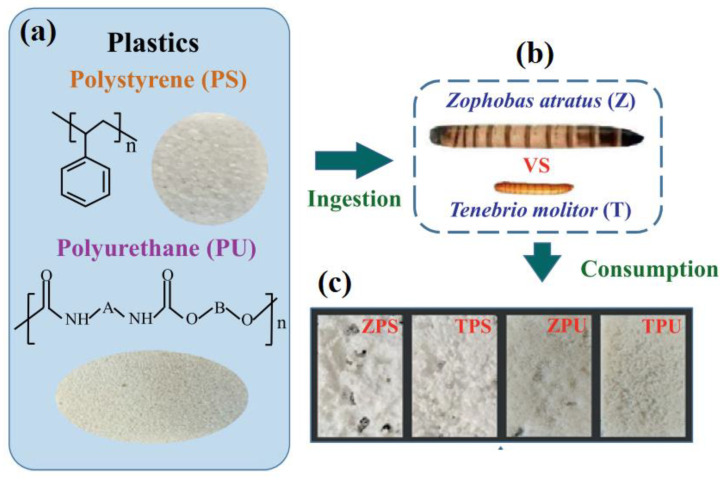
(**a**) PS and PU used in the study. (**b**) Hollows and pits on PS (**c**) *Zophobas atratus* and *Tenebrio molitor* used in the stud, adapted from [35].

Xin Zhao et al. fed *Z. atratus* and *T. molitor* with PS or PU foam plastic for 35 days and bran as the control. Figure 3a–c shows the PS, PU, *Z. atratus*, and *T. molitor* used in the study and hollows and pits on PS. The survival rate of *Z. atratus* was 100%, but weight loss was observed after 20 days of only using plastic. In contrast, the survival rates of *T. molitor* in the PS or PU groups were 84.67% and 62.67%, respectively, and the weight of the two groups increased. The accumulative consumption of plastic by the *Z. atratus* is 49.24 mg PS/larva and 26.23 mg PU/larva, which are 18 times and 11 times higher than those of *T. molitor*, respectively [35].

**Figure 4:**
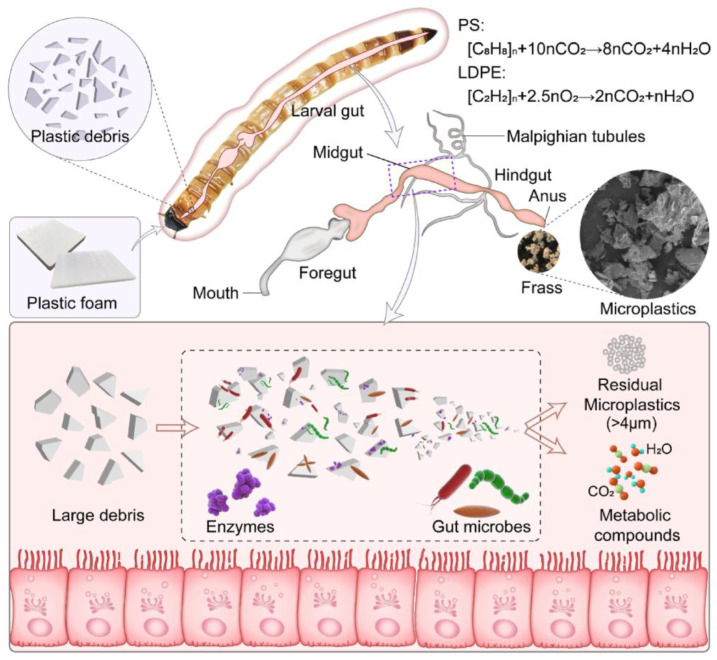
Conceptual schematic diagram for the biodegradation of PS and LDPE foams in *Zophobas atratus* larvae, reprinted with permission from [36].

Yalei Zhang et al. studied the fragmentation, larval physiology, intestinal microbiota, and microbial functional enzymes of ingested polymers through 28 days of experiments. Figure 4 shows the conceptual schematic for the biodegradation of PS and LDPE foams in *Z. atratus* larva. Larvae maintained a high survival rate, but when fed PS or LDPE, their body fat content decreased, and their consumption rates were 43.3 ± 1.5 and 52.9 ± 3.1 mg plastic/100 larvae/day, respectively. Ingested PS and LDPE were broken to an average size of 174 μM and 185 μM microplastics (by volume), size 6.3 μM and 5.9 μM particles reached the maximum number, respectively, and no nanoplastic was produced. This work provides new insights into insect-mediated biodegradation of persistent plastics for future research [36].

Dae-Hwan Kim et al. reported that *Pseudomonas aeruginosa* strain DSM 50071, which was isolated from the gut of *Z. atratus*, could degrade PS. Pseudomonas sp. DSM50071 could effectively biodegrade PS similarly to other plastic-degrading bacteria. The conversion of PS surface from hydrophobicity to hydrophilicity through biofilm formation is crucial for PS degradation [33].

Bryan J. Cassone reported that *Z. atratus* showed more microbial abundance in the early stage (24–72 h) of LDPE feeding than caterpillars fed with starvation or natural honeycomb diet. By using PS as sole carbon source to isolate and grow intestinal bacteria for more than one year, the microorganisms in Acinetobacter participated in the biodegradation process [37]. 

**Figure 5:**
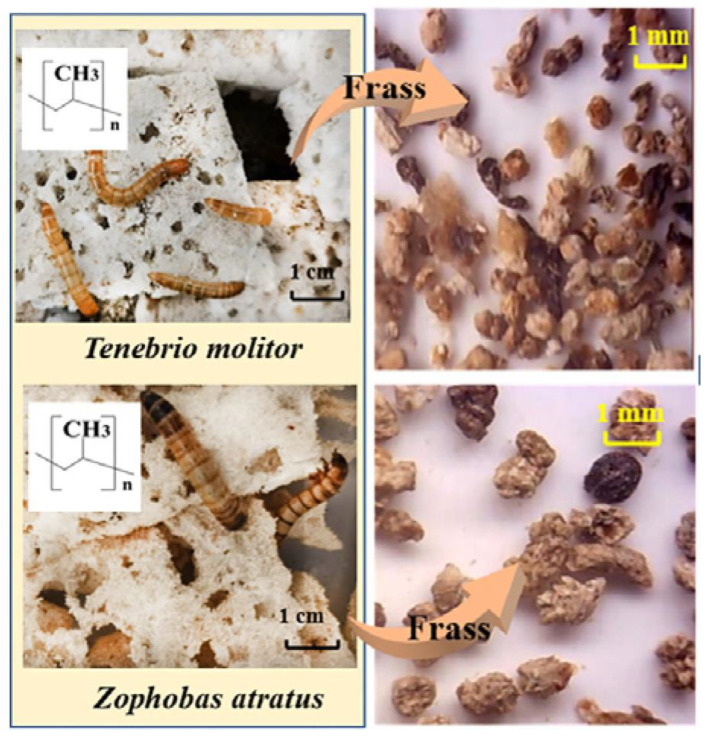
PP foam-eating *T. molitor* larvae and *Z. atratus* larvae, reprinted with permission from [38].

Shan-Shan Yang et al. investigated the biodegradation of PP by feeding larvae of *Z. atratus* and *T. molitor* with PP foam. Figure 5 shows the PP foam-eating *T. molitor* larvae and *Z. atratus* larvae, PP-fed versus PP + WB-fed gut microbiome of *Z. atratus* larvae. In the study, PP foam was used as sole diet as a comparative study. When larvae of *Z. atratus* and *T. molitor* were fed with the PP foam plus wheat bran, the consumption rates were enhanced by 68.11% and 39.70%, respectively. Mw of frass decreased by 20.4% and 9.0%, respectively, which indicated that PP can be biodegraded by the larvae of *Z. atratus* and *T. molitor* via gut microbe-dependent depolymerization [38].

Liping Luo et al. used plastics, including PS, PE and PU foam, as sole feedstock to feed *T. molitor*. PS- or PU-fed larvae showed 100% survival rates, and the PE-fed and starvation larvae had decreased survival rates of 81.67% and 65%, respectively. Plastic-fed and starvation groups showed decreased larvae weight. The consumption rates of PS, PE, and PU were 1.41, 0.30, and 0.74 mg/d/larva, respectively. The results showed that *T. molitor* can partially degrade plastics [34].

### 2.3. Plastic Degradation by G. mellonella

*G. mellonella* is a completely metamorphosed insect that undergoes four stages, including egg, larva, pupa, and adult. The insects are widely distributed all over the world, especially in the tropical and subtropical regions of Southeast Asia [51,52,53]. *G. mellonella* is rich in protein, short in growth cycle, and easy to reproduce, eat, and feed [54]. It is one of the main insect models which is used to study innate immunity and host pathogen interactions. The giant *G. mellonella* is often used to feed freshwater fish, birds, reptiles, and amphibians, which have three generations a year, and one generation lasts approximately 60–80 days [55]. When the average temperature exceeds 13 °C, the larvae begin to awake. The activity of the giant *G. mellonella* is closely related to external temperature. Excessively low or high temperature will slow the growth and even kill giant *G. mellonella*. *G. mellonella* likes to eat beeswax, and the chemical structure of the most common hydrocarbon bond in beeswax is highly similar to that of PE. The chewed PE is digested by the wax borer and converted into small molecules of ethylene glycol, which can be degraded in the natural environment within a few weeks.

**Figure 6:**
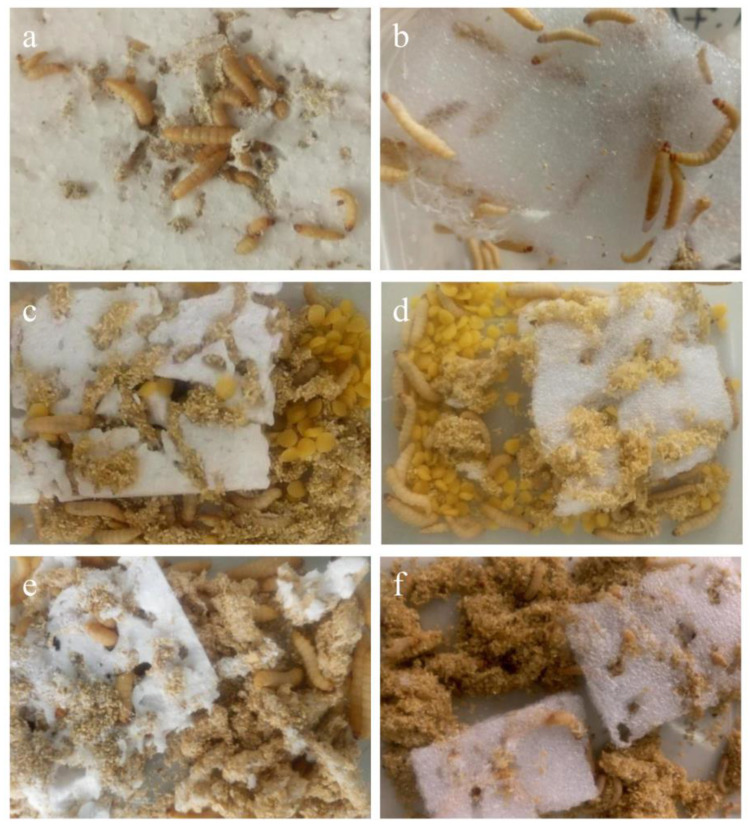
Plastic and supplementary nutrients were ingested by the greater wax moth larvae fed a PS diet (**a**), a PE diet (**b**), a PS + beeswax diet (**c**), a PE + beeswax diet (**d**), a PS + bran diet (**e**), and a PE + beeswax diet (**f**), reprinted with permission from [39].

Defeng Xing et al. investigated the feasibility of enhancing larval survival and the effect of supplementing the co-diet on plastic degradation by feeding the larvae of *G. mellonella*. Figure 6 shows that plastic and supplementary nutrients are ingested by the greater wax moth larvae fed with a PS diet. Significant mass loss of plastic was observed within 21 days (i.e., 150 larvae fed only PS or PE consumed 0.88 g and 1.95 g, respectively). O-containing functional groups and long-chain fatty acids are detected in metabolic intermediates, thereby showing depolymerization and biodegradation [39]

**Figure 7:**
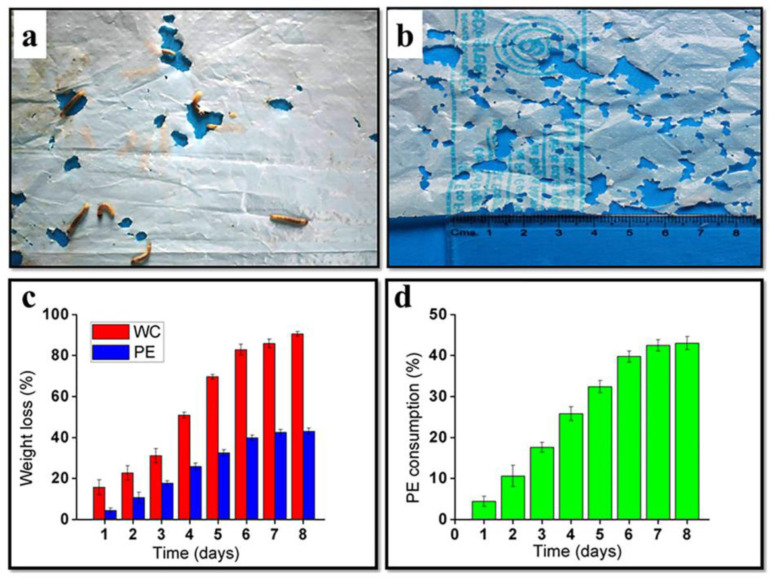
(**a**) *Galleria mellonella* larvae feeding on PE film. (**b**) Degraded PE film with holes after exposure to the lesser waxworm for 12 h. (**c**) Comparison of post-degradation weight loss percentage of waxcomb and PE after lesser waxworm consumption. (**d**) PE consumption over time, reprinted with permission from [40].

Harsha Kundungal et al. investigated the degradation of high-density polyethylene (HDPE) by feeding the larvae of *G. mellonella*. Nutrition on PE degradation were investigated by providing wax comb as co-feed. Figure 7a–d shows *G. mellonella* larvae feeding on PE film, degraded PE films with holes after exposure to the lesser waxworm for 12 h, comparison of post-degradation weight loss percentage of waxcomb and PE after lesser waxworm consumption, and PE consumption over time. The study after degradation showed that 100 wax insects reduced the weight of PE by 43.3 ± 1.6%. In 8 days, each wax insect ingested 1.83 mg of PE every day, and the consumption of PE increased [40].

**Figure 8:**
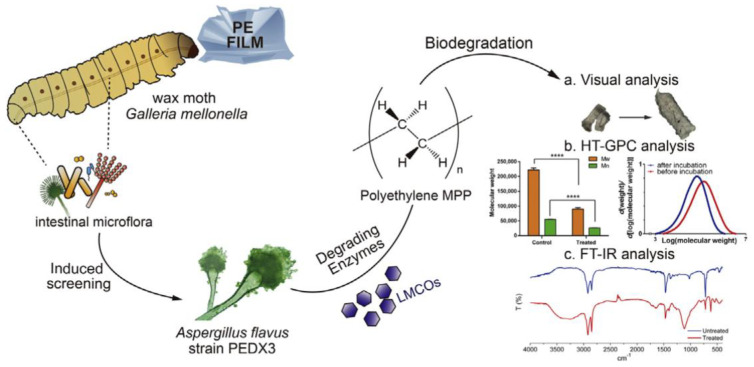
The process of PE films degraded by PEDX3(a PE degrading fungus) from the intestine of *Galleria mellonella*. (**a**) Visual analysis; (**b**) HT−GPC analysis; (**c**) FT−IR analysis, reprinted with permission from [41].

Yucheng Zhao et al. isolated a PE degrading fungus called PEDX3 from the intestine of *G. mellonella*. Figure 8a–c shows the process of PE films being degraded by PEDX3 from the intestine of *G. mellonella*, visual analysis, HT-GPC analysis, and Fourier infrared spectroscopy (FT-IR) analysis. The results showed that strain PEDX3 degraded HDPE and MPP into low molecular weight MPP after 28 days of culture. The degradation products contain carbonyl and ether groups, which verifies the degradation of PE [41].

Wei-Min Wu et al. investigated the biodegradability of LDPE and HDPE by yellow and dark *G. mellonella*. The sequence of biodegradation extent showed LDPE > HDPE. The low molecular weight, high branching, and low crystallinity of PE are positive for bio-degradation. Molecular weight is the key factor that affects biodegradability [42].

### 2.4. Plastic Degradation by Other Insects

Wax insects, which are mainly distributed in more than 10 provinces such as Shandong, Hebei, Henan, Sichuan, Yunnan, Guizhou, Guangxi, and Guangdong are special resource insects in China. They are also distributed in Japan, India, Russia, and other countries. The female only undergoes three stages, namely egg, nymph, and adult, which belongs to incomplete metamorphosis type. The male undergoes four stages, namely egg, larva, pupa, and adult, hence belonging to the completely changed S type. Guocai Zhang et al. studied the mechanism of PE degradation by wax insects. They fed the wax insects with PE, separated and purified two strains with high PE degradation efficiency, and evaluated the impact of single and microbial combination on PE degradation. The results showed that PE could be degraded by *Meyerospira gilsonii* and *Serratia marcescens*. However, the degradation efficiency of microbial community is higher, and the weight loss rate of PE is 15.87%. The chemical structures of a series of PE degradation products were obtained. This study can be used to develop an effective microbial community for PE degradation and provide a basis for the reuse of PE waste [56].

**Figure 9:**
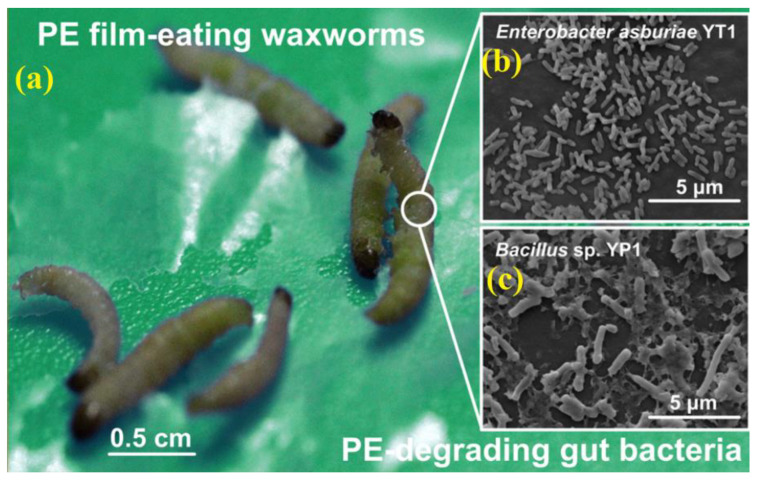
(**a**) PE film-eating waxworms. (**b**,**c**) Morphotypes of the cells in the mature biofilm on the PE sheet, reprinted with permission from [43].

Jun Yang et al. isolated two bacterial strains, namely *Escherichia coli* AST1 and Bacillus sp.YP1, which could degrade PE from the intestinal tract of wax worms. Figure 9a–c shows the PE film-eating waxworms and morphotypes of the cells in the mature biofilm on the PE sheet. After 28 days of culture on PE film, the two strains formed a living biofilm, the hydrophobicity of the PE film was reduced, and the obvious damage included pits and cavities. During the 60-day incubation period, YP1 could degrade by approximately 10.7 ± 0.2% of PE film. The molecular weight of the residual PE film is low, hence providing promising evidence for the biodegradation of PE in the environment [43].

**Figure 10:**
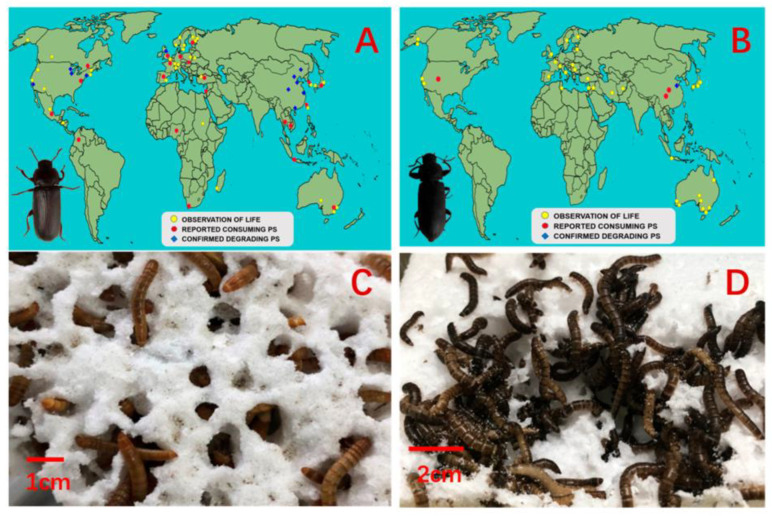
*Tenebrio molitor* and *Tenebrio obscurus* around the world and PS foam-eating behaviors. (**A**) Discovered life and original sources of *T. molitor*. (**B**) Discovered life and original sources of *T. obscurus*. (**C**) PS foam-eating *T. molitor* larvae and (**D**) *T. obscurus* larvae from Shandong Province, China, reprinted with permission from [44].

Yalei Zhang showed that the degradation rate of PS in the intestine of dark mealworms was faster than that of *T. molitor*. Figure 10A–D presents the *T. molitor* and *Tenebrio obscurus* around the world and PS foam-eating behaviors. With expanded PS foam as the only diet, after 31 days, Mn of residual PS in the feces of dark mealworms decreased by 26.03%, which was significantly higher than that of whitefly (11.67%). According to the proportion of PS residues, dark mealworms can degrade PS effectively [44].

Zhang Yong Wang et al. studied the biodegradation of PS in the intestinal microbiota of *T. molitor*, Parasita magna, and Atlas *Z. atratus* larvae. The results showed that the superworm had the strongest PS consumption ability and the highest survival rate during the 30-day experiment period, able to degrade PS to different degrees. *T. molitor* strongly depolymerized PS by destroying benzene ring [57].

Feng Ju et al. isolated a PVC-degrading bacteria from the intestinal tract of insect larvae and studied the pathway of PVC degradation. Their research reported that the larvae of the pest *Spodoptera frugiperda* can survive by eating PVC film, which is related to the enrichment of enterococcus, Klebsiella, and other bacteria in the larval intestinal microbiota. Bacterial strains isolated from larval intestine can depolymerize PVC [58].

Mik Van Der Borgt et al. investigated the effects of PVC plastics on the growth, survival, and biotransformation of black soldier fly larvae. The growth, survival, and biotransformation parameters of larvae were measured by feeding black soldier fly larvae with artificial food waste mixed with micro, medium, and large plastics. The insects are not affected by PVC plastics in the matrix in terms of growth performance, survival rate, and biotransformation rate [45].

**Figure 11:**
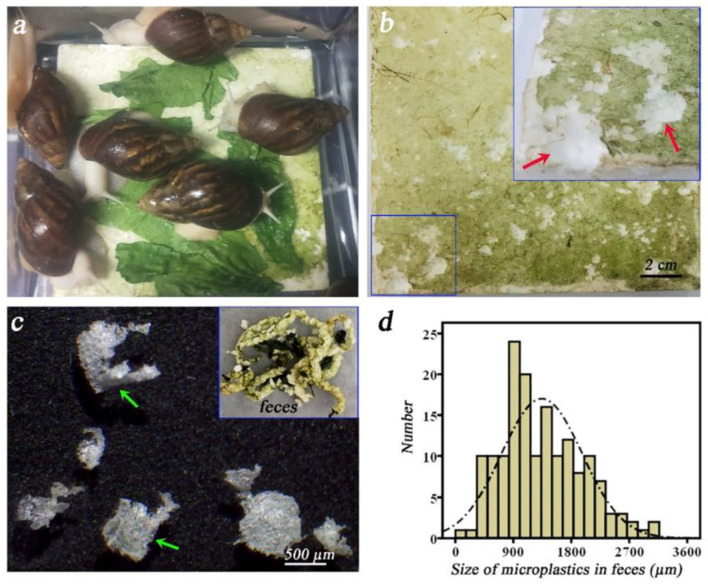
PS foam were uptaken by *A. fulica* and fragmented into microplastics. (**a**) The exposure scene of Styrofoam and *A. fulica*. (**b**) Mark of gnawing on Styrofoam (the top right showing enlarged image in the blue box). (**c**) Microplastics in feces. (**d**) The distribution of microplastic sizes, reprinted with permission from [59].

Defu He et al. reported the biodegradation capability of expanded PS foam in a globally distributed soil invertebrate, Achatina fulica. Figure 11a–d shows that the PS foam was uptaken by *A. fulica* and fragmented into microplastics. After a 4-week exposure, 18.5 ± 2.9 mg PS was ingested per snail in one month and microplastics in feces were egested with significant mass loss of 30.7%. A significant increase in Mw of feces-residual PS illustrated limited extent of depolymerization. Significant shifts in the gut microbiome were observed after the ingestion of PS, with an increase in families of Enterobacteriaceae, Sphingobacteriaceae, and Aeromonadaceae, which showed that gut microorganisms were associated with PS biodegradation [59].

Seongwook Woo et al. reported the PS biodegradation by the larvae of the darkling beetle *Plesiophthalmus davidis. P. davidis* ingested 34.27 mg of PS foam per larva and survived by feeding only on Styrofoam in two weeks. The ingested PS foam was oxidized. The decrease in the Mw of the residual PS in the frass compared with the feed PS foam, and C–O bonding was detected in the degradation products of PS film, which illustrated that PS foam was degraded [60].

## 3. Conclusions and Prospects

Plastic products have been widely used around the world because of its easy processing, low price, and portability. However, so far, unreasonable disposal of plastic products has led to serious pollution, and no perfect strategy can address waste plastics. It is difficult for plastics to degrade by themselves. Landfill has been proved to be infeasible for waste plastic disposal. Toxic gases are generated by incineration or chemical treatment, which cause various threats to the atmospheric environment and human health. Achieving large-scale disposal of waste plastics through recycling is also difficult. Insect-degradable plastics have not been employed in practical application. The current research reported that *T. molitor*, *Z. atratus*, wax borer, silkworm, and termite can feed on degradable plastics. Using insects to degrade plastics has the advantages of low cost and no secondary pollution. Moreover, the aforementioned insects can be used as animal feed sources, with good application and economic value. However, due to the differences in the composition and structure of plastics, the degradation efficiency of different insects varies greatly, and some plastics also affect the growth and development of insects. The research on plastic degradation by insects and isolated functional microorganisms has only been carried out in recent years, and the results are far from practical application. The researchers can conduct experiments in discovering more insects that can feed on plastic, confirming the preference of different insects for the types of plastic and further improving the efficiency of insects’ feeding and degradation of plastic. The use of waste plastics as food for insects can realize the recycling of waste plastics. However, the realization of this process requires researchers to conduct toxicological analysis on plastic-eating insects to avoid the toxicological risks or cumulative effects of toxic substances in organisms. High-throughput sequencing technology is used to analyze the intestinal microbial diversity of plastic-degrading insects, detect functional microorganisms related to plastic metabolism, reveal the realization mechanism of plastic microbial degradation and new metabolic path, and isolate and purify plastic-degrading functional microorganisms.

## Figures and Tables

**Table 1:** The latest works on degradation of polymer plastics by insects.

Polymer Plastic	References	Insect
LDPE foams	[23,24]	*Tenebrio molitor*
PS	[25,26,27]	
PE	[28]	
PVC	[29]	
Bio-based cross-linked polymer	[30]	
PU	[31]	
PS	[32,33,34]	*Zophobas atratus*
PU	[34,35]	
LDPE foams	[36,37]	
PP	[38]	
PS	[39]	*Galleria mellonella*
HDPE	[40]	
PE	[41]	
HDPE	[42]	
PE	[43]	Wax worm
PS	[44]	Dark mealworms
PVC	[45]	Black soldier fly larvae

## Data Availability

Not applicable.

## Abbreviations

PS	Polystyrene
PVC	Polyvinyl chloride
PE	Polyethylene
ABS	Acrylonitrile butadiene styrene
PU	Polyurethane
PLA	Polylactic acid
PHAs	Polyhydroxy fatty acid ester polymers
PPC	Carbon dioxide copolymer
PCL	Polycaprolactone
PGA	Polyglycolic acid
YT2	Exiguobacterium sp.
LDPE	Low-density PS
Mn	Molecular weight
THF	Tetrahydrofuran
FT-IR	Fourier infrared spectroscopy
ATR-FTIR	Fourier transform attenuated total reflection infrared spectroscopy
HDPE	High-density PS
